# Eicosapentaenoic and Docosahexaenoic Acid Levels in Mouse Tissues After Intake of Echium and Ahiflower Oils Rich in Stearidonic and α‐Linolenic Acids

**DOI:** 10.1002/lipd.70041

**Published:** 2026-02-13

**Authors:** Letícia V. Segre, Mariana S. Bisinotto, Inar A. Castro

**Affiliations:** ^1^ Department of Food and Experimental Nutrition, Faculty of Pharmaceutical Sciences, LADAF University of São Paulo São Paulo Brazil

**Keywords:** docosahexaenoic, eicosapentaenoic, mice, stearidonic, tissue distribution

## Abstract

Omega‐3 fatty acids (n‐3 FA), particularly eicosapentaenoic acid (EPA) and docosahexaenoic acid (DHA), have been consumed aiming to reduce cardiovascular disease. Fish oil is the main dietary source of EPA and DHA but has limitations that have stimulated interest in sustainable alternatives such as Echium ( *Echium plantagineum* ) and Ahiflower ( *Buglossoides arvensis* ) oils, both rich in α‐linolenic acid (ALA) and stearidonic acid (SDA), precursors of EPA. However, the ability of these two oils to increase EPA and DHA levels in different tissues remains unclear. Thus, this study aimed to investigate the effects of SDA‐rich oils on fatty acid profiles in different biological matrices of C57BL/6 mice. Animals received diets supplemented with soybean oil (control), Echium oil, or Ahiflower oil (4% diet) for 8 weeks, providing n‐3/n‐6 FA intake ratios of 0.14, 1.51, and 2.28, respectively. Fatty acids were analyzed in plasma, erythrocytes, liver, adipose tissue, heart, and brain. Both SDA‐rich oils significantly increased EPA levels across all tissues in a dose‐dependent manner compared with the control, whereas changes in DHA were limited and tissue‐specific. Despite the increase in EPA, DHA levels remained unchanged in the heart, brain, and plasma. In erythrocytes, DHA levels were higher in both SDA‐rich oil groups compared with the control. Echium and Ahiflower oils may be strategically used in EPA‐focused interventions and, depending on the target tissue and physiological demand, may partially satisfy DHA requirements. This nuanced understanding is critical for the development of evidence‐based dietary recommendations and sustainable omega‐3 supplementation strategies.

## Graphical abstract

Dietary supplementation with stearidonic acid–rich Echium and Ahiflower oils increases EPA levels across multiple mouse tissues, while DHA responses remain tissue‐specific and limited, supporting the strategic use of plant‐based omega‐3 sources for EPA‐focused interventions.

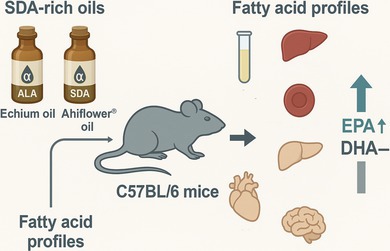

## 1 Introduction

The omega‐3 fatty acids (n‐3 FA) market, including supplements, fortified foods, infant formulas, pet food, and pharmaceuticals, reached globally USD 52.4 billion in 2023 (Global Organization for EPA and DHA Omega‐3s (GOED) 2024). One of the reasons for n‐3 FA supplement consumption is their ability to reduce inflammation, thereby contributing to the prevention and management of cardiovascular diseases (Calder 2017; Ridker 2016). Oxylipins, formed through the enzymatic oxidation of n‐3 FA, particularly eicosapentaenoic acid (EPA; 20:5 n‐3) and docosahexaenoic acid (DHA; 22:6 n‐3), exhibit anti‐inflammatory properties that help inflamed tissues return to homeostasis (Calder 2017; Serhan et al. 2008), becoming both, EPA and DHA, the main bioactive components responsible for the anti‐inflammatory effects.

EPA and DHA can be synthesized endogenously from α‐linolenic acid (ALA, 18:3 n‐3) or obtained directly from dietary sources or supplements, such as fish or algae oils. The synthesis of n‐6 and n‐3 polyunsaturated fatty acids (PUFA) begins with the essential precursors linoleic acid (LNA) and α‐linolenic acid (ALA), respectively, and involves the action of microsomal Δ6 desaturase (Δ6D), encoded by the fatty acid desaturase 2 (FADS2) gene; Δ5 desaturase (Δ5D), encoded by the fatty acid desaturase 1 (FADS1) gene; elongases 2 and 5 (encoded by the elongation of very long‐chain fatty acids genes ELOVL2 and ELOVL5); and peroxisomal β‐oxidation. Desaturation and elongation generally occur as alternating reactions, with the initial Δ6‐desaturation step considered rate‐limiting, whereas elongation reactions proceed more rapidly. Further conversion of EPA to DHA requires two additional elongation steps, forming docosapentaenoic acid (DPA, 22:5 n‐3) and tetracosapentaenoic acid (TPA, 24:5 n‐3), followed by a second Δ6‐desaturation to generate tetracosahexaenoic acid (THA, 24:6 n‐3) via the Sprecher pathway, and final peroxisomal β‐oxidation to yield DHA (Valenzuela et al. 2024, 2025; Videla et al. 2022). The conversion efficiency of ALA to EPA and DHA is very low, rendering this process highly inefficient (Lefort et al. 2016; Whelan 2009; Sayanova and Napier 2011; Baker et al. 2025; Ruiz‐López et al. 2012; Metherel et al. 2024; Burdge and Calder 2005; West et al. 2019). Estimated conversion rates are < 5%–10% for EPA and < 2%–5% for DHA (Lane et al. 2022; Saini et al. 2021). However, in a recent study, it was reported that the whole body DHA synthesis rates from dietary ALA is about 9.5% while in serum the observed rate was only 0.2% (Rotarescu et al. 2024), suggesting that the rate depends on the evaluated tissue.

Fish oil is the main source of EPA and DHA for human supplementation. However, it has notable drawbacks, including high cost, unpleasant sensory characteristics, cholesterol content, and potential contamination with environmental pollutants such as mercury, dioxins, and polychlorinated biphenyls. Additionally, concerns over the sustainability of global fish stocks are increasing (Ruiz‐López et al. 2012; Burdge and Calder 2005; West et al. 2019; Mozaffarian and Rimm 2006). Alternative n‐3 FA sources, such as plant‐derived oils, transgenic plants, and microalgae fermentation, are being investigated but still face challenges related to production costs, quality control, and sustainability (Ruiz‐López et al. 2012; West et al. 2019). Among these, plant‐based oils such as Ahiflower trademarked from *Buglossoides arvensis* and Echium ( *Echium plantagineum* ) represent potential alternatives for human supplementation. Both belong to the *Boraginaceae* family and are rich in stearidonic acid (SDA, 18:4 n‐3), which bypasses the rate‐limiting Δ6‐desaturation step in the n‐3 FA biosynthetic pathway, thereby enhancing the conversion efficiency to EPA and DHA when compared with ALA (Baker et al. 2025; Saini et al. 2021; EFSA Panel on Dietetic Products, Nutrition and Allergies (EFSA NDA Panel) 2015; Greupner et al. 2019).

The liver exhibits the highest capacity for PUFA biosynthesis, with more limited activity in the brain, testicle, and kidney, and no detectable activity in the heart or lung (Calder 2017; Valenzuela et al. 2024, 2025). This biosynthetic capacity is modulated by multiple factors, including sex, age, body mass index, dietary n‐6 FA, the n‐3/n‐6 ratio, genetic polymorphisms, hormonal changes, metabolic diseases such as type 2 diabetes and non‐alcoholic fatty liver disease (NAFLD), lifestyle factors (physical activity, smoking, and alcohol consumption), and overall nutritional status. Additional determinants include ALA availability; limited elongation and/or desaturation of DHA precursors; oxidative stress status; upregulation of Δ‐6 and Δ‐5 desaturase expression by insulin or activation under moderate food restriction and adequate protein intake; downregulation of these enzymes by glucagon, epinephrine, and glucocorticoids or inhibition by glycerol, low protein intake, or high consumption of fructose, glucose, cholesterol, trans fatty acids, trace elements (Zn^2+^, Mg^2+^, Ca^2+^), and vitamins (B3, B5, B6, and biotin); as well as DHA metabolism into bioactive docosanoids, including resolvins, maresins, and protectins, which actively participate in the resolution of inflammation (Valenzuela et al. 2024; Videla et al. 2022; Burdge and Calder 2005; Saini et al. 2021; Rotarescu et al. 2024; Greupner et al. 2019; Harnack et al. 2009).

Therefore, given their fatty acid profile, supplementation with Ahiflower or Echium oil is expected to promote a higher increase of EPA and possibly DHA than soybean oil. However, data quantifying these effects, particularly across different tissues, remain limited. In human studies, pharmacokinetic evaluation is generally based on blood samples, and it is still unclear whether blood or serum fatty acid levels accurately reflect changes induced by SDA‐rich oils in other tissues. Therefore, despite metabolic differences between humans and animal models (Valenzuela et al. 2024; Whelan 2009; Kawabata et al. 2013), it is important to investigate the capacity of SDA‐rich oils to increase EPA and DHA in other biological matrices.

Tissues differ in their capacity to accumulate fatty acids, largely as a consequence of differences in metabolic activity (Valenzuela et al. 2024). Chronic consumption of ALA has been shown to increase EPA concentrations in plasma and cells, with little or no corresponding increase in DHA (Burdge and Calder 2005). To the best of our knowledge, this is the first study to compare two different SDA‐rich oils within the same experimental design. Therefore, the objective of our study was to investigate whether consumption of SDA‐rich oils derived from Ahiflower and Echium seeds differentially increases ALA, SDA, EPA, and DHA across distinct biological samples. This information is essential to support evidence‐based recommendations by health agencies regarding sustainable alternatives to fish oil as sources of n‐3 FA.

## 2 Material and Methods

### 2.1 Materials

Soybean oil was purchased from a local supplier. *Echium plantagineum L*. oil (NEWmega Echium Oil, batch 08005) was provided by De Wit Speciality Oils (De Waal, Tescel, Netherlands) and contained 0.1% mixed tocopherols. Ahiflower oil ( *Buglossoides arvensis* , batch NAHI10538) was donated by Natures Crops International (York, UK) and stabilized with 0.1% rosemary extract (Fortium RPT40IP). Analytical standards were obtained from Sigma‐Aldrich (Sigma Chemical Co., St. Louis, MO, USA).

### 2.2 Study Design

**FIGURE 1:**
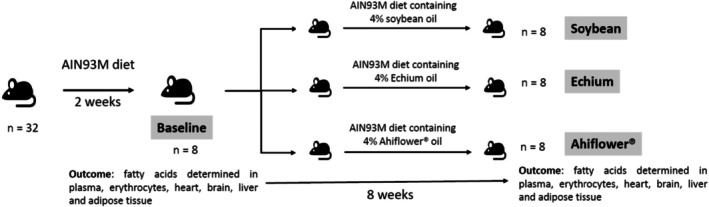
Experimental design.

Three‐month‐old male C57BL/6 NTac homozygous mice were acquired from the University of São Paulo. All experimental procedures were approved by the Institutional Animal Care and Use Committee of the Faculty of Pharmaceutical Sciences, University of São Paulo (CEUA/FCF 666/2023). Mice were housed in plastic cages (four animals per cage) under controlled conditions: temperature 25°C ± 2°C, relative humidity 55% ± 10%, and a 12‐h light–dark cycle. After a two‐week acclimation period with *ad libitum* access to the standard AIN‐93 M diet (Reeves et al. 1993), animals were randomly assigned to four groups (*n* = 8/group). The baseline group was euthanized after 2 weeks to characterize the parameters observed in the animals at the beginning of the study, while the remaining groups: SOYBEAN, ECHIUM and AHIFLOWER received modified AIN‐93 M diets for 8 weeks. The experimental design is shown in Figure 1. The modified diets contained 4% of refined soybean, Echium, or Ahiflower oil, respectively. Details on diet formulation and chemical composition are provided in Tables S1 and S2, respectively. At the end of the intervention, mice were fasted for 10 h, anesthetized with 3% isoflurane, and euthanized. Blood was collected for plasma and erythrocyte lipid profile analysis. The liver, adipose tissue, heart, and brain were excised, weighed, and stored at −80°C for further analyses.

### 2.3 Chemical Composition of the Diets and Oxidative Stability of the Oils

Diet composition was determined following AOAC methods (AOAC 2012). The oxidative stability of the oils was assessed by measuring lipid hydroperoxide (LOOH) concentrations (Shanta and Decker 1994). LOOH absorbance was read at 510 nm using a spectrophotometer (Synergy HT, BioTek, Winooski, VT, USA) equipped with Gen5 v1.06 software. A cumene hydroperoxide standard curve (1–30 μM) was used for quantification, with results expressed as meq O₂ kg^−1^ oil.

### 2.4 Tocopherol Composition of the Oils

The tocopherol content was determined by reversed‐phase high‐performance liquid chromatography (HPLC) method for determination of α‐, (β + γ), and δ‐tocopherol, using a HPLC (Agilent Technologies 1200 series, Santa Clara, CA, USA), equipped with a Zorbax Eclipse XDB‐C18 (150 mm × 4.6 mm; 5 μm) with pre‐column Eclipse XDB‐C18 4‐Pack Analytical Guard Column (12.5 mm × 4.6 mm; 5 μm) from Agilent Technologies. Oil samples (0.2 g) were diluted in 1.5 mL of 2‐propanol, vortexed, filtered, and injected into the HPLC system. A mobile phase was composed by 50% of acetonitrile and 50% methanol. The injection volume was 20 μL at the flow rate 1 mL/min. The eluate was detected with a fluorescent detector set at emission wavelength of 325 nm and excitation at 295 nm. Column temperature was kept at 35°C. Curves were prepared using corresponding standards from Sigma‐Aldrich (Alpha‐47,783, Delta‐47,784 and Gamma tocopherol‐47,785). Results were expressed as mg/100 g oil.

### 2.5 Fatty Acid Profiles of Oils, Diets, and Biological Samples

Lipids from biological samples, including plasma, erythrocytes, and tissues, were initially extracted using the Bligh & Dyer method (Bligh and Dyer 1959). After extraction, the lipids were hydrolyzed and then derivatized. Oils were directly weighed (5 mg). In brief, the oils and the extracted lipids were placed in tubes containing 20 μL of the internal standard (tricosanoic acid methyl ester, 5 mg/mL isooctane solution), 50 μL of a 0.5% BHT methanolic solution, and 2 mL of 0.5 M methanolic NaOH. The mixture was vortexed and heated in a water bath at 100°C for 5 min. After cooling, 2.0 mL of 14% BF3 in methanol was added, followed by vortexing and another 5‐min heating at 100°C, according to Shirai (Shirai et al. 2005). The mixture was cooled again, and 2 mL of isooctane was added. The tubes were shaken vigorously, followed by the addition of 5 mL of a saturated NaCl solution, and the tubes were gently homogenized. The organic upper phase was carefully transferred to another tube and dried under nitrogen. Finally, the dried lipid was resuspended in 300 μL of isooctane and transferred to an insert. The vial was injected into a gas chromatograph coupled with a single quadrupole mass spectrometer (GC–MS; Agilent 7890A GC System, Agilent Technologies Inc., Santa Clara, USA). Fatty acids were separated on a fused silica capillary column (J&W DB‐23, Agilent Inc., Santa Clara, USA), with dimensions of 60 m × 0.250 mm. A 1 μL injection volume was used in splitless, and the GC inlet temperature was set at 240°C. High‐purity helium was used as the carrier gas at a flow rate of 1.0 mL/min. The oven temperature was programmed to start at 80°C for 1 min, then increase at a rate of 10°C/min to 175°C. The temperature was then raised by 4°C/min to 230°C and held for 5 min. The transfer line temperature was maintained at 280°C. Mass spectra were obtained using electron impact ionization (70 eV) in scan mode (35–550 m/z). Fatty acids were identified by comparing the retention times of samples with those of standards (FAME 37 Component Mix Supelco 47,885), as well as by matching mass spectra with the National Institute of Standards and Technology (NIST) spectral database (NIST, Gaithersburg, MD, USA). All analyses were conducted in duplicate. Fatty acid composition was expressed as relative percentage of total identified fatty acids, calculated from the ratio between the peak area of each fatty acid methyl ester and that of the internal standard (tricosanoic acid methyl ester, C23:0). Because the aim of the analysis was to determine fatty acid profiles rather than absolute concentrations, individual calibration curves for each fatty acid were not applied. This approach assumes comparable detector response among fatty acid methyl esters under electron impact ionization conditions (70 eV), which is widely accepted for compositional analyses of fatty acids in biological samples.

### 2.6 Statistical Analysis

Data were analyzed using one‐way ANOVA followed by Tukey's HSD test when variance was homogeneous, or Kruskal‐Wallis ANOVA when heterogeneity was detected. Correlation between the fatty acids intake and their concentration after the trial was calculated based on simple regression. Results are expressed as mean ± SD, with *p* < 0.05 considered statistically significant. Statistical analyses were performed using Statistica v13 (TIBCO Software Inc., Palo Alto, CA, USA). Graphs were prepared using GraphPad Prism (GraphPad Software, San Diego, CA, USA).

## 3 Results

**FIGURE 2:**
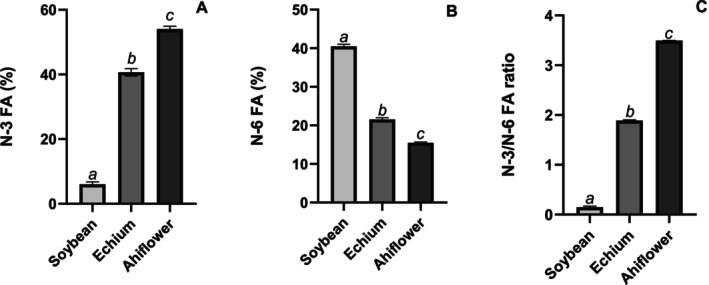
Proportion of fatty acids observed in the oils: N‐3 FA (A), n‐6 FA (B) and n‐3/n‐6 FA ratio (C). Data are mean ± SD; bars showing different superscript letters represent statistically significant difference between the groups (*p* < 0.05).

Figure 2 presents the n‐3 FA, n‐6 FA, and n‐3/n‐6 FA ratio of the oils applied in this study. It was observed that all oils differed, increasing the n‐3 FA content from soybean to Echium and Ahiflower oil. Fatty acid profile, tocopherol content, and hydroperoxide concentration of the oils are shown in Table 1. The fatty acid profiles of the three oils used in this study were consistent with values previously reported in the literature and established databases (Lefort et al. 2016, 2017; Carlini et al. 2021; Nogueira et al. 2016; Government of Canada 2024; Bisinotto et al. 2022). Additionally, the oxidative stability of the oils, evaluated via lipid hydroperoxide (LOOH) concentrations, was within the acceptable range for refined oils (LOOH < 10 meq O_2_/kg), as defined by the *Codex Alimentarius* Commission (Codex Alimentarius Commission 2023). As expected for vegetable oils, γ‐tocopherol was the predominant tocopherol isomer across all samples, and total tocopherol content was similar to levels reported in previous studies (Nogueira et al. 2016; Bisinotto et al. 2022). These results confirm the quality and authenticity of the oils used in this experiment.

**TABLE 1:** Fatty acid profile, tocopherol content and hydroperoxide concentration of the oils.

	OILS^a^		
	Soybean	Echium	Ahiflower
Fatty acids (%)			
C14:0—Myristic acid	0.20 ± 0.06	—	—
C16:0—Palmitic acid	19.92 ± 0.70	13.37 ± 0.27	11.75 ± 0.57
C16:1—Palmitoleic acid	0.24 ± 0.03	—	0.12 ± 0.05
C17:0—Heptadecanoic acid	0.09 ± 0.01	0.13 ± 0.03	0.06 ± 0.00
C17:1 cis—Heptadecenoic acid	0.04 ± 0.00	—	0.05 ± 0.00
C18:0—Stearic acid	7.91 ± 0.42	9.67 ± 1.62	7.58 ± 1.19
C18:1 n‐9 cis—Oleic acid	24.46 ± 0.80	13.97 ± 0.34	10.17 ± 0.62
C18:2 n‐6—Linoleic acid (LNA)	40.46 ± 0.56	12.75 ± 0.28	10.83 ± 0.15
C18:3 n‐6—γ‐Linoleic acid (GLA)	—	8.76 ± 0.20	4.63 ± 0.09
C18:3 n‐3—α—Linolenic acid (ALA)	6.04 ± 0.76	28.21 ± 0.90	37.91 ± 0.61
C18:4 n‐3—Stearidonic acid (SDA)	—	12.47 ± 0.27	16.13 ± 0.26
C20:0—Arachidic acid	0.30 ± 0.06	0.05 ± 0.09	—
C20:1 n‐9 cis—Eicosenoic acid	0.06 ± 0.06	0.62 ± 0.01	0.78 ± 0.02
C22:0—Behenic acid	0.26 ± 0.27	—	—
C22:1 n‐9—Erucic acid	—	—	—
Tocopherol (mg/100 g oil)			
α Tocopherol	4.66 ± 0.01	4.33 ± 0.10	1.54 ± 0.07
γ Tocopherol	63.29 ± 1.31	77.93 ± 1.95	54.78 ± 0.83
δ Tocopherol	6.04 ± 0.13	4.41 ± 0.12	2.12 ± 0.04
Oxidative Marker			
Hydroperoxides (meq O_2_/Kg)	2.34 ± 0.15	1.15 ± 0.06	1.22 ± 0.06

^a^ Values are expressed as mean ± SD.

Diet intake did not differ among groups (Table 2); however, lipid intake was slightly lower in Echium and Ahiflower groups (*p* = 0.009) when compared with the Soybean group. The estimated daily intake of ALA and SDA increased from the Soybean to the Echium and Ahiflower group (*p* < 0.001). Final body weight was greater in echium‐fed mice compared with soybean‐fed animals (*p* = 0.048), while Ahiflower showed intermediate values.

**TABLE 2:** Body and tissue weight and diet intake of groups.

	Groups^4^				*p* ^2^	*p* ^3^
	Baseline	Soybean	Echium	Ahiflower^1^		
Body weight						
Initial weight (g)	20.58 ± 0.94	20.66 ± 1.28	20.88 ± 1.30	21.03 ± 1.03	*0.855*	—
Final weight (g)	24.74 ± 1.69	28.19 ± 2.31^a^	31.25 ± 2.77^b^	29.17 ± 1.88^ab^	—	*0.048*
Body weight gain (%)	—	36.39 ± 5.97^a^	49.53 ± 7.45^b^	38.69 ± 6.60^a^	—	*0.002*
Time of the trial (days)	—	56	55	54		
Tissues weight						
Spleen (mg)	57.14 ± 3.65	69.08 ± 6.37	74.93 ± 8.55	74.36 ± 10.20	—	*0.336*
Liver (mg)	982.28 ± 137.85	959.61 ± 93.26	1019.23 ± 119.60	914.61 ± 98.25	—	*0.157*
Heart (mg)	118.56 ± 13.61	121.86 ± 10.37	125.14 ± 10.45	117.64 ± 9.99	—	*0.360*
Brain (mg)	311.63 ± 31.63	405.38 ± 19.20	375.66 ± 65.11	366.39 ± 53.84	—	*0.223*
Diet intake (g/day)	—	2.88 ± 0.09	2.98 ± 0.10	2.91 ± 0.04	—	*0.544*
Lipid intake (g/day)	—	0.16 ± 0.01^a^	0.14 ± 0.00^b^	0.12 ± 0.00^b^	—	*0.009*
ALA intake (mg/day)^5^	—	9.14 ± 0.29^a^	37.89 ± 1.30^b^	47.27 ± 0.58^c^	—	*< 0.001*
SDA intake (mg/day)^5^	—	0.00 ± 0.00^a^	16.75 ± 0.58^b^	20.11 ± 0.25^c^	—	*< 0.001*
ALA total intake (g)^5^	—	0.51 ± 0.02^a^	2.08 ± 0.07^b^	2.55 ± 0.03^c^	—	*< 0.001*
SDA total intake (g)^5^	—	0.00 ± 0.00^a^	0.92 ± 0.03^b^	1.09 ± 0.01^c^	—	*< 0.001*

^1^ Values expressed in mean ± SD. Different superscript letters represent statistically significant difference between the groups (*p* < 0.05).

^2^ Probability value obtained by One way ANOVA followed by Tukey's test among the four groups.

^3^ Probability value obtained by one‐way ANOVA followed by Tukey's test among the groups except BASELINE.

^4^ BASELINE: group euthanized at the beginning of the assay.

^5^ Values estimated from data shown in Table 1.

**FIGURE 3:**
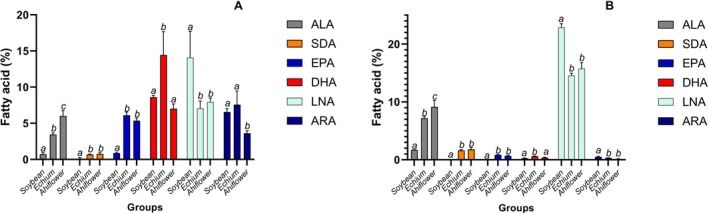
ALA, SDA, EPA, DHA, LNA and ARA proportion observed in the liver (A) and adipose tissue (B) after 8 weeks of supplementation. Data are mean ± SD; Data are mean ± SD; bars showing different superscript letters represent statistically significant difference between the groups (*p* < 0.05); ns means not significant.

**FIGURE 4:**
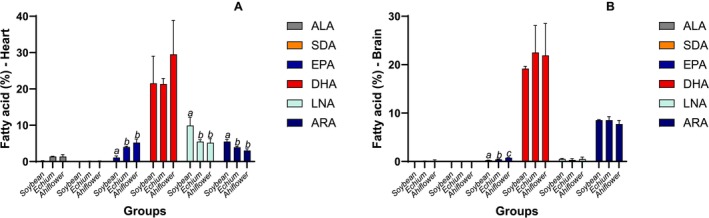
ALA, SDA, EPA, DHA, LNA, and ARA proportion observed in the biological samples after 8 weeks of supplementation in the heart (A) and brain (B). Data are mean ± SD; Data are mean ± SD; bars showing different superscript letters represent statistically significant difference between the groups (*p* < 0.05); ns means not significant.

**FIGURE 5:**
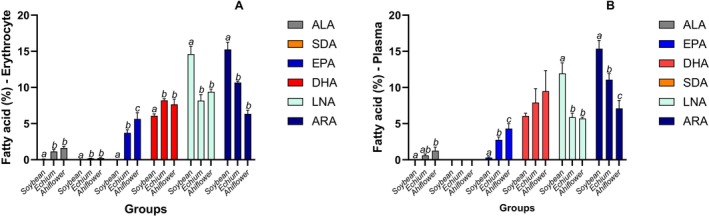
ALA, SDA, EPA, DHA, LNA and ARA proportion observed in the biological samples after 8 weeks of supplementation in the liver erythrocytes (A) and plasma (B). Data are mean ± SD; Data are mean ± SD; bars showing different superscript letters represent statistically significant difference between the groups (*p* < 0.05); ns means not significant.

The effects of the different oils on major n‐3 and n‐6 FA proportion varied according to the biological matrix (Figures 3, 4, 5; Tables S3–S7). Echium and Ahiflower oils increased hepatic ALA, SDA, and EPA relative to Soybean oil (*p* < 0.05). DHA proportion was higher in the Echium group than in the Soybean and Ahiflower groups (Figure 3A). The fatty acid peaks were identified using mass spectrometry, excluding any possibility of identification mistake, since the DHA peak was perfectly isolated in the chromatogram (Figure S1). Echium and Ahiflower oils decreased LNA compared with Soybean oil, but only Ahiflower oil reduced ARA in the liver (Figure 3A).

In adipose tissue, both SDA‐rich oils increased ALA, SDA, and EPA compared with soybean oil, while DHA increased only in the Echium group (Figure 3B). Both Echium and Ahiflower oils decreased LNA and ARA compared with soybean oil. Regarding the heart (Figure 4A), Echium and Ahiflower oils increased only EPA, and both decreased LNA and ARA compared with soybean oil. The effects of oil supplementation in the brain were observed only for EPA (Figure 4B). In erythrocytes (Figure 5A), Echium and Ahiflower oils increased all the major n‐3 FA analyzed and also reduced both n‐6 FA, LNA, and ARA. A similar result was observed in plasma, except for the absence of SDA and no effect on DHA (Figure 5B).

**FIGURE 6:**
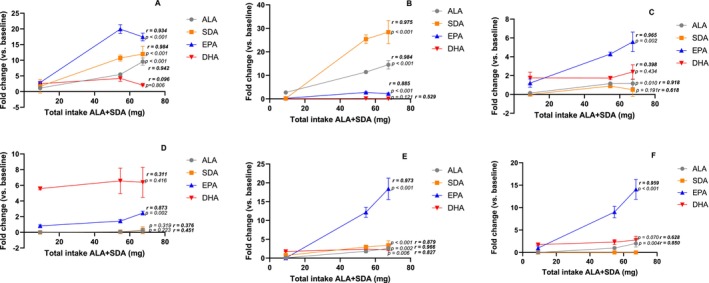
Fold change (vs. baseline) according to total ALA + SDA intake after 8 weeks of supplementation (Table 2) for the proportions of ALA, SDA, EPA, and DHA observed in biological samples: (A) Liver; (B) Adipose tissue; (C) Heart; (D) Brain; (E) Erythrocyte and (F) Plasma. Data are presented as mean ± SD. Linear regression coefficient (*r*) and probability value (*p*).

Finally, the correlation between the total ALA+ SDA intake and the change of n‐3 FA from their respective baseline levels was evaluated in all biological samples (Figure 6). In the liver (Figure 6A) and adipose tissue (Figure 6B), it was observed that there was a linear correlation between the total ALA+ SDA intake and ALA, SDA, and EPA fold change from baseline. In the heart (Figure 6C) and brain (Figure 6D), there was correlation only to EPA. All n‐3 FA increased according to the increase of total ALA+ SDA intake in the erythrocytes (Figure 6E), while in plasma (Figure 6F), just ALA and EPA showed significant correlation.

Across all biological matrices, SDA‐rich oils consistently increased EPA, with a limited and tissue‐specific effect on DHA. Notably, Echium oil increased hepatic and adipose DHA, while both Echium and Ahiflower oils increased erythrocyte DHA. These findings support the use of SDA‐rich plant oils as effective dietary sources for elevating EPA, with more restricted potential to increase DHA.

## 4 Discussion

In the present study, the consumption of 0.16 g/day, 0.14 g/day and 0.12 g/day of Soybean, Echium and Ahiflower oils respectively, represented a different intake of ALA + SDA, increasing from Soybean (9.14 mg/day) to Echium (54.64 mg/day) and Ahiflower (67.38 mg/day) groups (Table 2). This difference promoted an increase of EPA and DHA that varied according to the biological sample analyzed, confirming the hypothesis of this study. In general, the low proportion of ALA and SDA found in almost all analyzed samples, even after supplementation, is due to the fact that the major metabolic fate of ALA appears to be oxidation, while SDA is rapidly converted to EPA (Whelan 2009; Harnack et al. 2009).

It was observed (Figure 3A) that Echium and Ahiflower oils increased ALA, SDA, EPA, and only Echium oil increased DHA in the liver. This increase occurs at the expense of LNA and ARA as reported in another study (Cardoso et al. 2018). This result is expected, since the liver has the highest capacity for PUFA biosynthesis (Valenzuela et al. 2025). However, while the increase of EPA was similar between the Echium and Ahiflower groups, the DHA proportion was the highest in the Echium group. In human hepatocytes, it was found that formation of EPA and DHA was highest at an LNA/ALA ratio of 1:1 (Harnack et al. 2009). However, the LNA/ALA ratio in the diet of the Echium and Ahiflower groups was very similar, 0.5:1 and 0.3:1 respectively, compared with 7.0:1 in the Soybean group, not justifying this unexpected result. Thus, other factors, such as those cited in previous studies (Valenzuela et al. 2024; Videla et al. 2022) could have influenced the conversion from EPA → DPA → TPA → THA → DHA in the mammalian “*Sprecher”* pathway, since the EPA proportion was similar in both groups fed with Echium or Ahiflower oils (Figure 3A). Although in a lower amount compared with the liver, a similar result was observed in the adipose tissue (Figure 3B). Our data also suggest that adipocytes are not adequate cells for sampling in studies involving n‐3 FA supplementation, since adipose tissues mainly accumulated LNA (Figure 3B).

It was observed a linear correlation between ALA + SDA intake and EPA enhancement in all tissues, whereas results for DHA were limited only to erythrocytes (Figure 6). Our results agree with a previous study in which an SDA‐soybean oil was applied to describe a dose‐dependent increase in EPA and DPA in serum and liver of rats by the intake of SDA, while only a slight response was found to DHA (Kawabata et al. 2013).

Heart (Figure 4A) and brain (Figure 4B) present the highest proportion of DHA esterifying the phospholipids in membranes. We found that in both these tissues, Echium and Ahiflower oils were able to increase EPA proportion compared with soybean oil, without any effect on DHA. Valenzuela et al. (2014) reported that hepatic DHA may result from its active transformation from ALA; meanwhile, brain DHA may be associated with the almost selective transport from the liver rather than being formed from ALA, considering that the concentration and activity of elongases and desaturases are much lower in the brain than in the liver.

Similarly, other human or animal studies have demonstrated that chronic consumption of ALA or SDA, either as pure encapsulated fatty acids or as components of oils, increases EPA concentrations in plasma and cellular pools, while conversion to DHA is minimal and non‐linear (Lefort et al. 2016; Burdge and Calder 2005; Greupner et al. 2019; Couëdelo et al. 2022). The inefficient capacity of ALA and SDA supplementation to increase DHA has been attributed to many factors. It has been reported that Δ6‐desaturase has a low affinity for its longer‐chain substrates, TPA and THA, which likely contributes to the limited conversion of EPA to DHA (Greupner et al. 2019). In addition, the rate‐limiting step of PUFA metabolism relies on the second reaction of Δ‐6D (Valenzuela et al. 2025). Couëdelo et al. (Couëdelo et al. 2022) suggested that increases in dietary ALA lead to an initial rise in tissue DHA followed by a plateau, indicating a non‐linear response. In vitro studies also report a saturation point for diet‐induced PUFA incorporation into cellular membranes, suggesting that the administration of n‐3 FA beyond a certain level suppresses formation of DHA from EPA (Harnack et al. 2009). Data from Whelan et al. (Whelan et al. 1991) propose a limited responsiveness of tissue DHA to increasing dietary ALA, consistent with a plateau‐like behavior at relatively low dietary n‐3/n‐6 ratios. Importantly, similar saturation behavior has been observed in dose–response studies using preformed DHA rather than ALA, as reported by Broughton et al. (Broughton et al. 1991), indicating that tissue DHA levels plateau even when conversion steps are bypassed. Collectively, these findings suggest that the capacity of tissues to incorporate and maintain DHA within membrane phospholipids is inherently limited, likely reflecting homeostatic regulation of membrane composition and unsaturation. Thus, the observed plateau in tissue DHA enrichment appears to be driven more by physiological constraints on tissue incorporation than by inefficiencies in the metabolic conversion of n‐3 precursors. Metherel et al. (Metherel et al. 2019) using compound‐specific isotope analysis (CSIA) by GC‐isotope ratio mass spectrometry (IRMS) concluded that the increase in plasma EPA following DHA supplementation in humans does not occur via retroconversion, but from a slowed metabolism and/or accumulation of plasma EPA. It has also been reported that rats are able to synthesize DHA from ALA at sufficient rates to supply the brain's daily DHA requirements (Baker et al. 2025; Cardoso et al. 2018). Our data indicate that the same condition could occur in the heart. Therefore, it can be suggested that Echium and Ahiflower oils at the dose applied in our study could provide enough ALA+SDA to maintain the needs in high‐priority tissues, such as brain and heart, as reported in a recent review (Baker et al. 2025).

To the best of our knowledge, this study is the first to compare Echium and Ahiflower oils in the same experimental design. Soybean oil was added to the design as a control, since AIM‐93 M diet (Reeves et al. 1993), recommend soybean oil to prepare the diet. Anyway, further studies could include other ALA‐rich oils options, such as chia, perilla, sacha inch, or flaxseed. It has been reported that fatty acids profiles determined in the erythrocytes reflect more chronic changes, while in plasma represent acute changes in diet and metabolism (Hu et al. 2017). Our data showed that fatty acids changes observed in the erythrocytes were more pronounced than in plasma, confirming that erythrocytes may represent a better matrix for evaluating fatty acids.

## 5 Conclusion

Our results indicate that dietary supplementation with ALA and SDA derived from Echium and Ahiflower oils, at the doses applied in our model, effectively increased EPA proportions in all analyzed tissues. The EPA increase due to Echium and Ahiflower oils intake was similar in liver, adipose tissue, and heart, whereas in brain, plasma, and erythrocytes, it was more pronounced after Ahiflower supplementation compared with Echium oil. The conversion to DHA showed a different pattern according to each tissue. Its levels remained unchanged in heart, brain and plasma, increased in liver and adipose tissue only after Echium supplementation, and were higher in erythrocyte in the groups fed with Echium and Ahiflower oils. Thus, Echium and Ahiflower oils may be strategically used in EPA‐focused interventions and, depending on the target tissue and physiological demand, may partially satisfy DHA requirements. This nuanced understanding is critical for the development of evidence‐based dietary recommendations and sustainable omega‐3 supplementation strategies.

## Author Contributions

Letícia V. Segre: Conceptualization, Methodology, Formal analysis, Investigation, Writing – original draft. Mariana S. Bisinotto: Writing – review and editing. Inar A. Castro: Conceptualization, Methodology, Resources, Writing – review and editing, Visualization, Supervision, Project administration, Funding acquisition. Authors used ChatGPT 4.0 to improve the readability of the manuscript. After using this tool, the authors reviewed and edited the content as needed and take full responsibility for the content of the published article.

## Funding

This study was supported by the Coordination for the Improvement of Higher Education Personnel—Brazil (CAPES), CNPq 303016/2024–8 and The State of São Paulo Research Foundation (FAPESP; grant 2023/07321–9 and 2023/14268–7).

## Ethics Statement

Trial procedures were approved by the ethics committee of the Faculty of Pharmaceutical Sciences, University of São Paulo. CEUA/FCF 063.2023. Approved on November, 9th 2023.

## Conflicts of Interest

The authors declare no conflicts of interest.

## Supporting information


**Figure S1:** DHA peaks observed in the chromatograms obtained from the liver after 8 weeks of supplementation in the Soybean group (A), Echium group (B), and Ahiflower group (C).
**Figure S2:** DHA peaks observed in the chromatograms obtained from the adipose tissue after 8 weeks of supplementation in the Soybean group (A), Echium group (B), and Ahiflower group (C).

## Data Availability

Data will be made available on request.
