# Supplementary material for: Eicosapentaenoic and Docosahexaenoic Acid Levels in Mouse Tissues After Intake of Echium and Ahiflower Oils Rich in Stearidonic and α‐Linolenic Acids
Source: Lipids. 2026 Feb 13;61(3):417–26. doi: 10.1002/lipd.70041 (PMC13144718; doi:10.1002/lipd.70041)
Supplement: Supplementary file 1 — Figure S1: DHA peaks observed in the chromatograms obtained from the liver after 8 weeks of supplementation in the Soybean group (A), Echium group (B), and Ahiflower group (C). Figure S2: DHA peaks observed in the chromatograms obtained from the adipose tissue after 8 weeks of supplementation in the Soybean group (A), Echium group (B), and Ahiflower group (C). [file LIPD-61-417-s001.docx]

| **A** |
| --- |
| **** |
| **B** |
| **** |
| **C** |
| **** |

**Supplementary Figure 1.** DHA peaks observed in the chromatograms obtained from the liver after 8 weeks of supplementation in the Soybean group (**Supp.Fig.1A**), Echium group (**Supp.Fig.1B**), and Ahiflower® group (**Supp.Fig.1C**).

| **A** |
| --- |
| **** |
| **B** |
| **** |
| **C** |
| **** |

**Supplementary Figure 2.** DHA peaks observed in the chromatograms obtained from the adipose tissue after 8 weeks of supplementation in the Soybean group (**Supp.Fig.2A**), Echium group (**Supp.Fig.2B**), and Ahiflower® group (**Supp.Fig.2C**).
